# List of Diseases at the Bath City Dispensary, from November 1, to December 1, 1801

**Published:** 1802-01

**Authors:** 


*List of Diseases at the Bath City Dispensary, from November* 1, *to December* 1, 1801.

| | | | |
|---|---|---|---|
| Angina | 1 | Petechia fine Febre | 1 |
| Anafarca | 6 | Pleurodyne | 2 |
| Contufion | 1 | Pfora | 2 |
| Catarrh | 5 | Rheumatifm Acute | 5 |
| Chlorofis | 1 | Synochus Biliofa | 23 |
| Diarrhœa | 2 | ———— Cholerica | 1 |
| Eryfipelas | 1 | ———— Dyfenterica | 2 |
| Enterodynia | 1 | ———— Enteritica | 1 |
| Febris Intermittens | 1 | Syphilis | 2 |
| Gaftrodynia | 7 | Suppuration | 1 |
| Hæmatemefis | 1 | Strain | 2 |
| Menorrhagia | 1 | Tumor | 1 |
| Peripneumonia Notha | 1 | Tinea Capitis | 1 |
| Pertuffis | 1 | Variola | 1 |
| Phthifis Pulmonalis | 4 | Ulcerated Leg | 1 |
| Pulmonary Complaint | 1 | Vermes | 1 |

It will appear from the above ftatement, that the Bilious Fever has decreafed fince the laft report. In fome inftances Typhus has evidently been combined with it. At prefent, pulmonary complaints are the moft prevalent.

*Bath, December* 11, 1801.

